# Winter connectivity and leapfrog migration in a migratory passerine

**DOI:** 10.1002/ece3.9769

**Published:** 2023-02-01

**Authors:** Rafael Rueda‐Hernández, Christen M. Bossu, Thomas B. Smith, Andrea Contina, Ricardo Canales del Castillo, Kristen Ruegg, Blanca E. Hernández‐Baños

**Affiliations:** ^1^ Museo de Zoología, Departamento de Biología Evolutiva, Facultad de Ciencias Universidad Nacional Autónoma de México Mexico City Mexico; ^2^ Colorado State University Fort Collins Colorado USA; ^3^ Center for Tropical Research and Institute of the Environment and Sustainability University of California Los Angeles California USA; ^4^ Department of Ecology and Evolutionary Biology University of California Los Angeles California USA; ^5^ Facultad de Ciencias Biológicas, Ciudad Universitaria, San Nicolás de los Garza Universidad Autónoma de Nuevo León Nuevo León Mexico

**Keywords:** ecology, full‐cycle, genoscape, leapfrog migration, *Passerina ciris*, stopover, winter ecology

## Abstract

Technological advances in migratory tracking tools have revealed a remarkable diversity in migratory patterns. One such pattern is leapfrog migration, where individuals that breed further north migrate to locations further south. Here, we analyzed migration patterns in the Painted Bunting (*Passerina ciris*) using a genetic‐based approach. We started by mapping patterns of genetic variation across geographic space (called a genoscape) using 386 individuals from 25 populations across the breeding range. We then genotyped an additional 230 samples from 31 migration stopover locations and 178 samples from 16 wintering locations to map patterns of migratory connectivity. Our analyses of genetic variation across the breeding range show the existence of four genetically distinct groups within the species: Eastern, Southwestern, Louisiana, and Central groups. Subsequent assignment of migrating and wintering birds to genetic groups illustrated that birds from the Central group migrated during the fall via western Mexico or southern Texas, spent the winter from northeastern Mexico to Panama, and migrated north via the Gulf Coast of Mexico. While Louisiana birds overlapped with Central birds on their spring migratory routes along the Gulf Coast, we found that Louisiana birds had a more restricted wintering distribution in the Yucatan Peninsula and Central America. Further estimation of the straight‐line distance from the predicted breeding location to the wintering location revealed that individuals sampled at lower winter latitudes traveled to greater distances (i.e., the predicted breeding area was further north; *p* > .001), confirming that these species exhibit a leapfrog migration pattern. Overall, these results demonstrate the utility of a genoscape‐based approach for identifying range‐wide patterns of migratory connectivity such as leapfrog migration with a high degree of clarity.

## Graphical abstract

We studied migration in the Painted Bunting (*Passerina ciris*) using genetic data. We found four genetically distinct groups: Eastern, Southwestern, Central, and Lousiana. *Passerina ciris* has a leapfrog migration pattern.

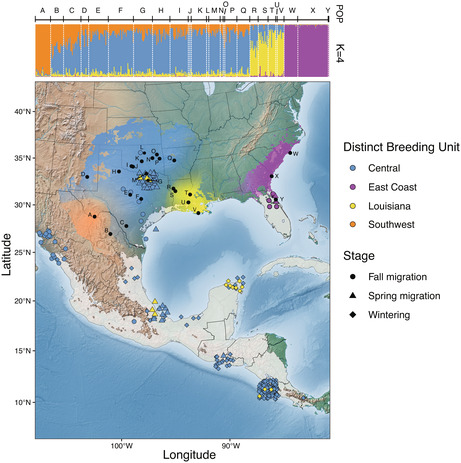

## 1 INTRODUCTION

Long‐distance migration allows birds to exploit resources differently across time and space and is one of their most intriguing behaviors. The assumption is that migration occurs because the conditions in the breeding range become adverse, and species move to regions where conditions are more favorable, and food is more abundant (Newton, 2008). Long‐distance migration patterns are varied across species depending on competition and resource availability across the annual cycle (Alerstam, 2003). Migratory connectivity describes the spatial and temporal links of individuals and populations between the seasons that result from the migratory movements carried out by organisms. Sometimes patterns of migratory connectivity are strong, in which case individuals from the same breeding population migrate to similar wintering regions, while sometimes migratory connectivity is weak, in which case individuals from similar breeding populations mix on their wintering grounds (Webster et al., 2002). Quantifying patterns of migration across the annual cycle is important not only for increasing our understanding of the ecology and evolution of migratory species (Winger et al., 2018; Winger & Pegan, 2021) but can help inform full life cycle conservation efforts for populations that are experiencing declines (Webster et al., 2002).

**FIGURE 1:**
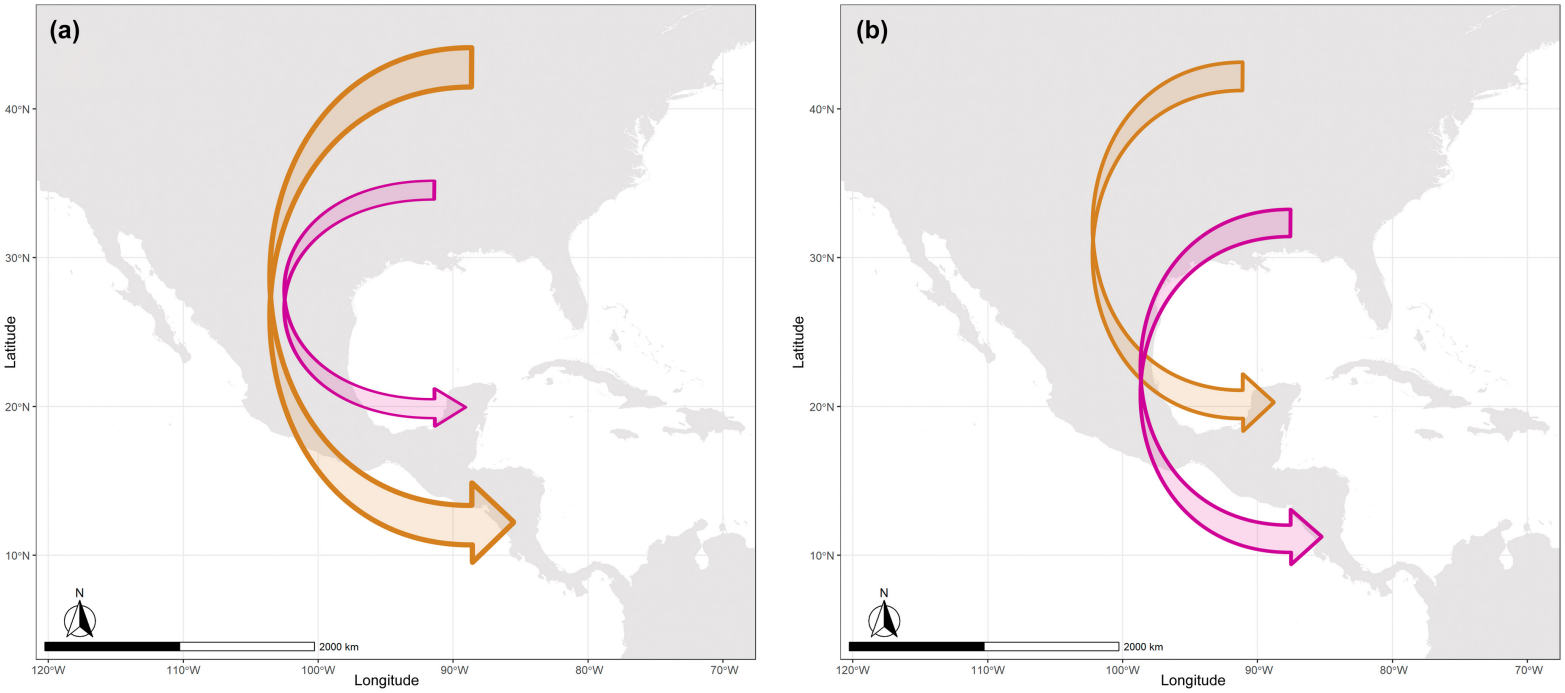
Typical migration patterns. (a) Leapfrog migration pattern. (b) Chain migration pattern.

One classic pattern of migration, which has been described, but only rarely documented with clarity, is leapfrog migration. This occurs when the most northerly breeding populations of a species winter the furthest south, while the populations that breed further south migrate a shorter distance to winter (Welty, 1982). As a result, most northerly breeding populations “jump over” these middle‐latitude populations during their migration (Figure 1). By contrast, the chain migration pattern occurs when the most northerly breeding populations winter migrate in the northernmost part of the winter range with the southernmost breeding populations winter further south (Figure 1; Fort et al., 2012). There are several classic examples of leapfrog migration across different species of birds, including Fox Sparrows, Wagtails, and Eastern Golden eagles (e.g., Åkesson et al., 2012, 2020; Boland, 1990; Nelson et al., 2015; Ramos et al., 2015), but the ability to detect patterns of leapfrog migration can be limited by the need for methods that quantify migratory connectivity across vast regions of the breeding and wintering range.

While in some cases improvements in tracking technology have increased our ability to detect leapfrog migration, in other cases, they have cast doubt on previous work. For example, direct measurements of individual birds' migrations using light‐level geolocators cast doubt on what is perhaps one of the most well‐documented cases of the leapfrog migration in nature (Fraser et al., 2018)—that of Fox Sparrow's breeding along the Pacific Coast of North America (Bell, 1997; Swarth, 1920). Early hypotheses proposed that leapfrog migration in the Fox Sparrow resulted from greater to access to resources in northern breeding sites outweighing the costs of longer migratory journeys to more southernly wintering locations. This hypothesis was supported by evidence of greater fat loads in more northerly breeding populations (Alaska) and lower fat loads in the populations that migrate shorter distances (Bell, 1997). However, geolocator tracks of northern and southern wintering populations of the Sooty Fox Sparrow (*Passerella ilica unalaschcensis*), did not support the leapfrog pattern. In contrast to expectations, individuals wintering further south overlapped on the breeding grounds with individuals that wintered further north and there was unexpectedly high degree of mixing between the two wintering ranges (Fraser et al., 2018). Thus, while patterns of leapfrog migration have been documented previously, there is a need for studies that can be used to comprehensively assess the frequency of this pattern in other migratory species.

A genetic framework that investigates migratory connectivity across the full annual cycle in 100–1000 of individuals has some advantages to studies that can provide highly detailed tracking information in one or two focused areas. The implementation of genetic methods often starts with the construction of a genoscape—a map of genetic variation across geographic space (sensu Ruegg et al., 2014)—which can be used as a baseline for assigning individuals from across the annual cycle back to their most likely breeding region. Once the genoscape has been constructed, genetic assays can be developed for specific SNPs allowing high‐throughput amplification of DNA even when it is highly fragmented or when only a small amount of DNA is available, as is often the case when DNA is obtained from feathers. The resulting genetic assays can be used to screen 100–1000 of individuals from outside of the breeding range, allowing for a comprehensive assessment of broadscale migratory connectivity patterns if sufficient samples are available. A genoscape‐based approach not only has the advantage of being able to comprehensively assess migratory patterns but can help with defining Conservation Units, which assists with the downstream management of a species across its full annual cycle.

The Painted Bunting is a migrant species that breeds in central and southeastern North America (Lowther et al., 2020; Tonra & Reudink, 2018) and winters from northwestern Mexico to Central America. Within the species, there are two recognized allopatric subspecies showing different molting and migratory strategies (Lowther et al., 2020; Thompson, 1991). The eastern subspecies (*ciris*) are distributed from North Carolina to Florida. The western subspecies (*pallidor*) has a distribution area that is 25 times larger than the eastern subspecies, which ranges from northeastern Mexico (Tamaulipas, Nuevo León, and Coahuila) to central Texas, southeast Kansas, east to west Tennessee, Mississippi and Oklahoma (Sharp, 2021). Recent population genetic structure studies have consistently revealed three populations based on the breeding range: one in North Carolina, South Carolina, Florida, and Georgia (Eastern breeding population), a second in Mississippi, Louisiana, Eastern and Central Texas (Central breeding population), and a third in Western Texas, Oklahoma and Arkansas (Western breeding population; Battey et al., 2018; Contina et al., 2019). Although the species has been well studied in most of its breeding range (i.e., the part of its range that falls within the USA), the genetic contributions of the Western breeding populations in northeastern Mexico have been neglected.

In this study, we used a genetic approach to investigate migration patterns in the Painted Bunting across time and space. We first combined RAD‐sequencing data (Contina et al., 2023) with targeted SNP genotyping data from 386 birds across the breeding range to create a breeding genoscape for the species. We then used SNP assays to genotype an additional 178 individuals from 16 wintering locations and 230 individuals from 31 stopover locations to assign each individual to distinct breeding genetic clusters and track the movement patterns in Painted Buntings across time and space. Finally, we used these combined data to explore migratory patterns in the Western breeding population and assess the potential for leapfrog migration.

## 2 METHODS AND MATERIALS

### 2.1 Sample collection and DNA extraction

We compiled a collection of 794 Painted Bunting blood and feather samples (see full metadata for samples in Table S1. Field collections permit from Instituto Nacional de Ecologia, SEMARNAT, Mexico were FAUT‐0169 and SGPS/DGVS/01595) provided by the Bird Genoscape project (https://www.birdgenoscape.org), collaborators from universities in Latin American countries, the Institute for Bird Populations, and independent banding stations. One hundred and twenty‐six individuals from 13 populations were previously used to test for genome‐wide population structure (Contina et al., 2017). In comparison, an additional 260 individuals spanning 25 breeding populations (12 new populations) were used to fill in sampling gaps and re‐assess the population structure. All samples were extracted using QIAGEN DNeasy Blood and Tissue Kits (San Francisco, CA). Blood extractions were further quantified using Qubit dsDNA HS157 Assay kits (ThermoFisher Scientific) and visually inspected via gel electrophoresis.

### 2.2 SNP‐type assay design and feather screening

From initial genome‐wide RAD‐sequencing for the Painted Bunting (see methods within Contina et al., 2017), we used *vcftools* (Danecek et al., 2011) to identify highly divergent SNPs that can be used to diagnose the four major genetic clusters identified in previous population genomic analyses (Contina et al., 2017). We used custom R scripts to create low‐cost SNP‐type assays from these initial divergent variants list. We used the R package *snps2assays* (Anderson, 2017) to evaluate which top‐ranking SNPs would generate designable assays for each genetic group. We characterized primers as designable if GC content was <0.65, there were no insertions or deletions (indels) within 30 bp, and there were no additional variants within 20 bp of the targeted variable site. Additionally, we aligned 25 bp surrounding the target variable site to the genome using bwa (Burrows‐Wheeler Aligner; Li & Durbin, 2009) to determine whether the designable primers mapped uniquely to the reference genome of a closely related relative, the Medium Ground finch, *Geospiza fortis* (Parker et al., 2012; NCBI Assembly ID: 402638 (GeoFor_1.0)), and to filter out those that mapped to multiple locations. We used this subset of primers to develop a SNP‐type assay (Fluidigm Inc.) that was used to screen 260 additional breeding individuals from across the breeding range and 408 individuals collected from wintering and migratory stopover sites for assignment to the breeding population of origin. Our wintering ground sampling in the west was exhaustive and ranged from northern Mexico to Costa Rica; however, our sampling did not include wintering sites in the east where the Eastern birds are known to overwinter (i.e., south Florida, the Bahamas, or Cuba; Robertson & Woolfenden, 1992; Raffaele et al., 1998; Rushing et al., 2021).

To screen the 66 designable assays of highly divergent variants, genotyping was performed on the FluidigmTM 96.96 IFC controller. We used the Juno GT Preamp Master Mix (Fluidigm, Item #100‐8363) for the preamplification of the SNPs and the Juno GT Preamp Master Mix for the final amplification. For each run, we screened 94 individuals and two nontemplate controls. We imaged the results on an EP1 Array Reader and called alleles using Fluidigm's automated Genotyping Analysis Software (Fluidigm Inc.) with a confidence threshold of 90%. In addition, we visually inspected all SNP calls and removed any calls that did not fall clearly into one of three clusters (heterozygote or either homozygote cluster). As DNA quality can affect call accuracy, we employed a stringent quality filter and dropped variants with missing calls exceeding 20%.

### 2.3 Genetic screening and building the genoscape

To create a genoscape with the best quality data, we filtered out samples with high values of relatedness to eliminate closely related samples from further analyses. To do this, we conducted a kinship analysis by formatting our whole genome data in the PLINK package (Purcell et al., 2007) for analysis with the KING toolset (Manichaikul et al., 2010). KING facilitates the assessment of genome‐wide paired associations or familiar relationships of large datasets. We excluded parents‐offspring (kinship value ~0.4) and first‐degree relatives (>0.18).

To assess the number of population clusters across the breeding range of each species, we used the Evanno method (Evanno et al., 2005) to determine the optimal number of genetic clusters identified using STRUCTURE (version 2.3.4; Pritchard et al., 2000). Although model‐based approaches describe continuous patterns of variation using discrete clusters, and may therefore overestimate the number of discrete clusters present, they are useful for addressing our overarching objective of describing the maximum number of genetically unique groups. Therefore, we implemented the *locprior* model, in which sampling populations are set to a specific distinct genetic cluster a priori. We ran 5 iterations of each assumed number of genetic clusters (*K*), where *K* ranged from 1 to 5 (Pritchard et al., 2000) and summarized the posterior probability of group membership estimates from the best structure run.

To create the Painted Bunting genoscape based on our highly divergent SNP‐type assays, we visualized the posterior probability of group membership estimates from STRUCTURE (Pritchard et al., 2000) as transparency levels of different colors overlaid on a base map from Natural Earth (naturalearthdata.com) and clipped to the Painted Bunting breeding range using the latest eBird shapefile (eBird, 2021) using the R packages sp, RGDAL, and raster (Bivand et al., 2013, 2017; Hijmans, 2019). We scaled the transparency of colors within each distinguishable group so that the highest posterior probability of membership in the group according to the structure was opaque and the smallest was transparent.

### 2.4 Baseline distinct genetic groups and accuracy of assignment

We evaluated the accuracy of individual assignments to each of the genetically distinct groups identified in the genoscape using self‐assessment testing in *rubias* (Moran & Anderson, 2018), a Bayesian hierarchical genetic identification approach that accounts for population structure and differences in the number of populations. The self‐assessment function in *rubias* tests the accuracy of assignment in terms of the proportion of individuals from known genetically distinct breeding groups that are assigned back to the correct breeding group. We considered a robust assignment as >0.8 posterior probability of assignment to the inferred group. We considered assignments with a posterior probability of <0.8 as uncertain and removed those individuals from the final reporting. Thus, reported misassignments refer to significant assignments in the wrong collection.

### 2.5 Assignment of unknown migratory and wintering birds

To determine the breeding origin of migrating and wintering birds, we utilized two methods. First, we assigned individuals collected from wintering and migratory stopover locations (i.e., whose breeding location was unknown—hereafter, “unknown” birds) to the genetically distinct groups characterized by the final genoscape using *rubias* (Moran & Anderson, 2018). We defined the unknown birds first by the mixture collection that corresponded to the location where they were collected and treated it as a separate sample group that gets its own mixing proportion estimate, and then by combining all unknown birds into one “mixture” category. Migratory and wintering individuals with a probability of assignment to a distinct genetic group >0.8 are reported. To provide a more fine‐scale estimate of breeding location compared with assignment to a distinct genetic cluster that can encompass a large geographic space, we subsequently estimated the geographic origin of individuals based on their genetic backgrounds using the R package *OriGen* (Ranola et al., 2014). The *OriGen* model divides the region of interest into pixels; in this case, the Painted Bunting breeding range generates allele frequency surfaces for each SNP and then applies Bayes' rule to compute the posterior probabilities to localize the origin of a given individual (Ranola et al., 2014). The pixel with the highest posterior probability is inferred to be the predicted geographic location of an individual. We then used the R program *geosphere* (v1.5‐10, Hijmans, 2019) to calculate the distance between the predicted breeding location and the known sampling location of wintering individuals.

## 3 RESULTS

### 3.1 Painted bunting population structure

**FIGURE 2:**
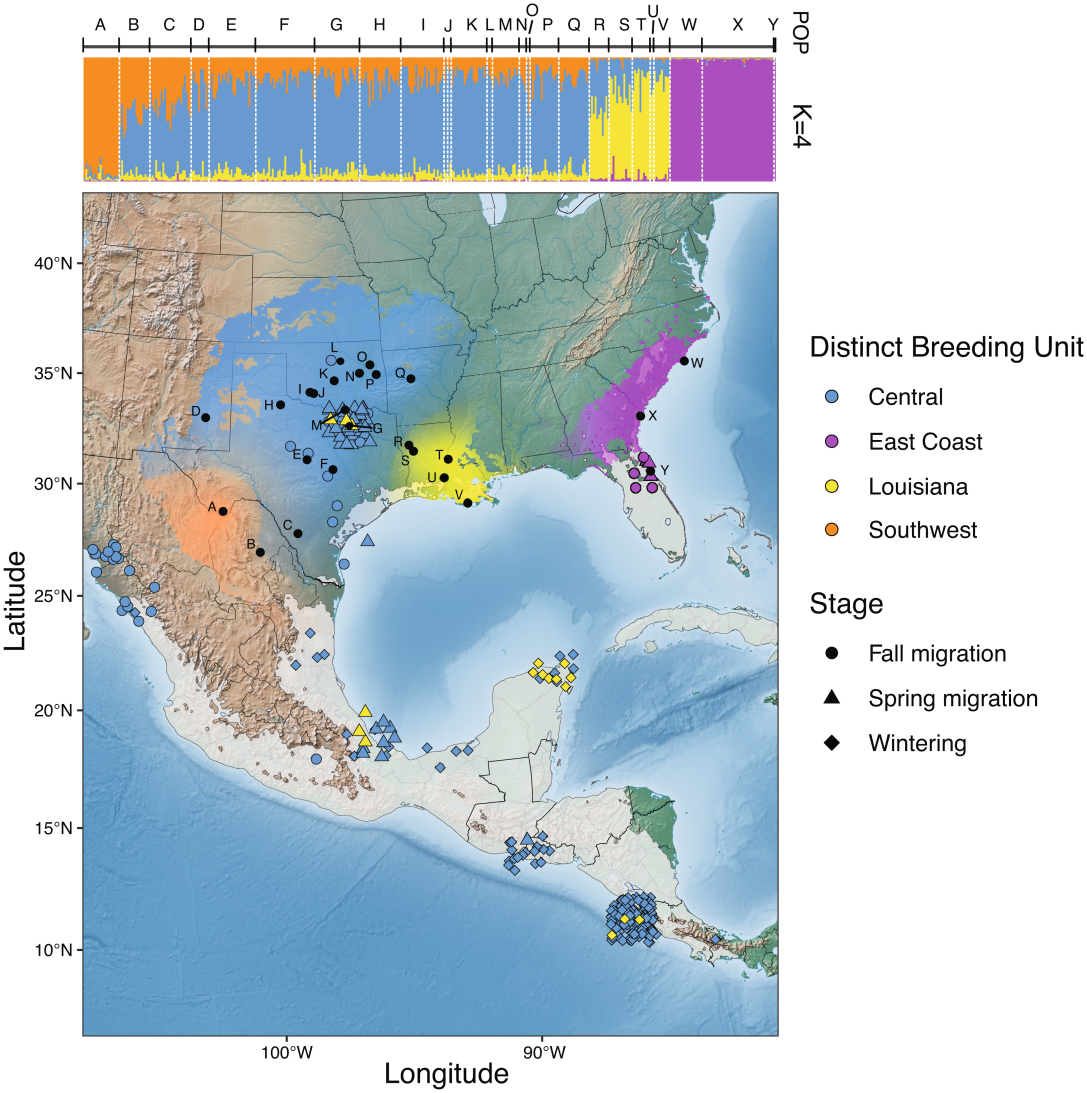
Top: Q matrix calculated from *locprior* model in STRUCTURE (*K* = 4). Orange depicts the Southwestern genetic group, blue depicts the Central genetic group, yellow depicts the Louisiana genetic group, and purple depicts the Eastern genetic group. Bottom: Breeding genoscape and assignment of migrant and wintering individuals of the Painted Bunting. Capital letters refer to the sampling locations from Table S1 on the breeding ground, and the transparent gray represents the wintering range.

Our STRUCTURE analysis of 386 samples from 25 populations across the breeding range showed that the breeding populations could be robustly divided into four genetically distinct groups: an eastern cluster represented by sampling in North Carolina, Georgia, and Florida, a southwest genetic cluster (Big Bend, TX), a coastal south‐central cluster in Louisiana, and a central cluster that ranges from an isolated New Mexico population to eastern Arkansas (Figure 2; Figure S1). Not surprisingly, the sampling locations that were most genetically distinct (i.e., did not demonstrate admixed ancestry with surrounding populations) were also the most geographically distant from each other: Big Bend (southwestern Texas) and the eastern populations, including Bald Head Island (North Carolina), Isle of Hope (Georgia), and Ormond Beach (Florida). In general, genetic distinctness increased with geographic distance throughout the sampled range, except for locations in Louisiana, which, despite being close to other sampling locations, had a very well‐differentiated genetic structure (Figure 2).

### 3.2 Breeding origin of migratory and wintering birds

Most birds captured outside the breeding season were assigned to the Central group (Figure 2). Ultimately, birds from the Central group migrate during the fall to western Mexico or southern Texas, winter in central and southern Mexico and Central America, and migrate back to their breeding grounds via the coastal plains of the Gulf of Mexico. The birds assigned to the Louisiana group were found to winter in the Yucatan Peninsula and Central America and migrate north during the spring along the coastal plains of the Gulf of Mexico (Figure 2). Of the birds sampled in the East, we were only able to sample birds during the spring and fall migration period, and all were assigned to the Eastern genetically distinct population (Figure 2). Unexpectedly, none of the individuals captured outside the breeding season were assigned to the Southwestern group, suggesting that either we did not sample where these birds winter or our primers could not distinguish the Central and Southwestern genetic cluster fully. The former is more likely given we demonstrate clear admixture between the Southwestern and Central genetic groups (Figure 2), and three birds collected from the Southwest breeding cluster were misassigned to the Central genetic cluster (Table S2). It is important to note that Big Bend, TX, the only population with nearly 100% Southwest ancestry, is isolated in the sky island of the Chisos Mountains (Heald, 1951; MacCormack et al., 2008). By contrast, the two populations closest to Big Bend, are on the eastern edge of these mountains and have mixed ancestry, suggesting these mountains represent a significant barrier to gene flow. Thus, the underlying admixture might limit the ability to assign admixed southwestern birds, and the location of the wintering Big Bend population remains unsampled.

**FIGURE 3:**
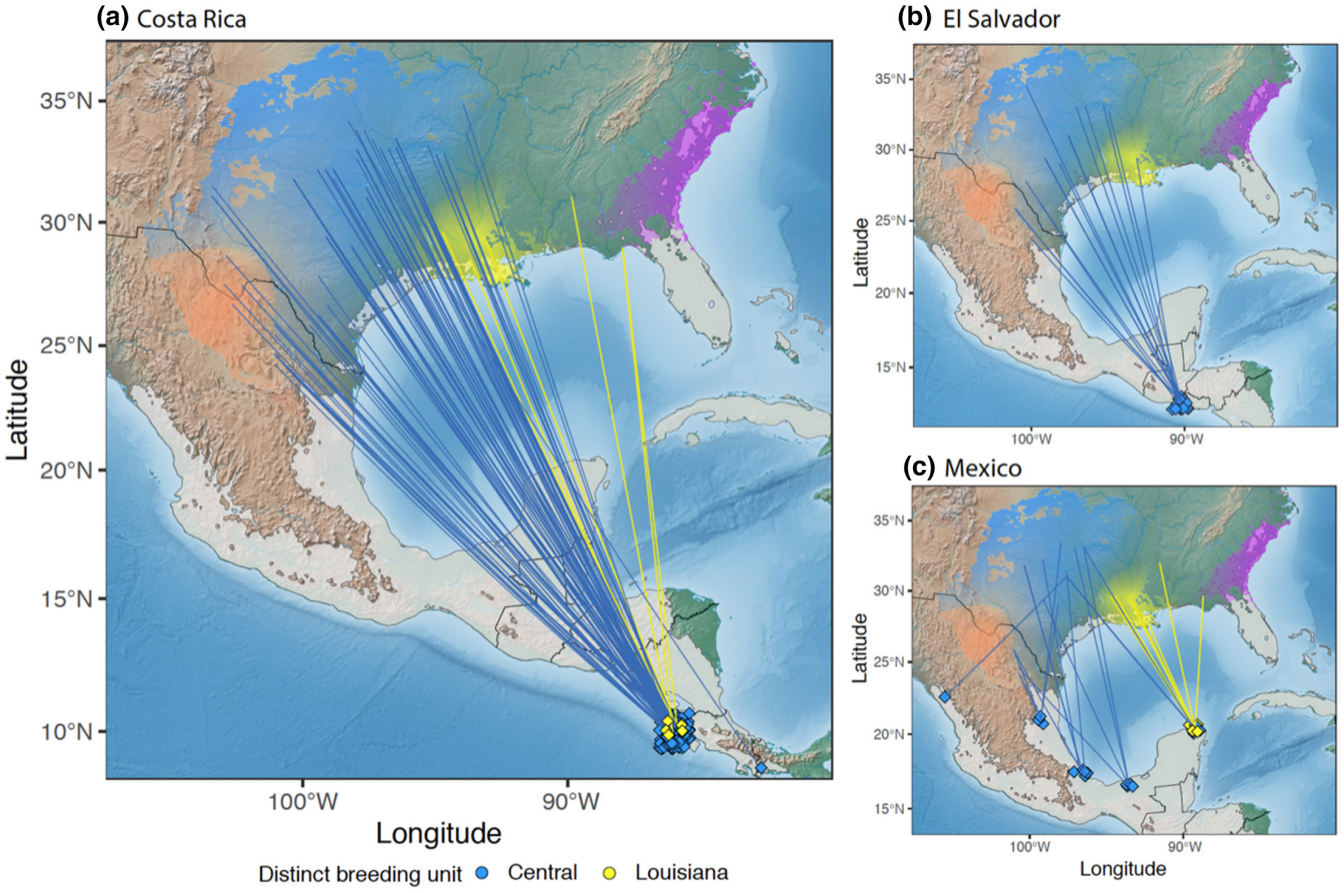
Predicted breeding locations of wintering birds sampled in (a) Costa Rica, (b) El Salvador, and (c) Mexico. Line segment colors represent the assignment to the breeding unit from *rubias* and spans from where the individual was collected on the wintering ground to the predicted breeding geographic location estimated in *OriGen*.

The predicted breeding location of wintering individuals supports the population assignments for the most part, as most wintering birds had predicted locations within the distribution of the Central genetic group (Figure 3). However, birds wintering on the Yucatan Peninsula had predicted breeding locations that included Louisiana and northern Florida, eastern Texas, Oklahoma, and Arkansas. Surprisingly, birds wintering in Costa Rica (the location with the most samples) predicted breeding locations from three genetically distinct groups, contrary to the genetic breeding unit assignment, which assigned all birds but one that winters in Costa Rica to the Central genetic cluster. Approximately 80% of the birds were predicted to be from the Central breeding group, followed by the Southwestern breeding group, and three individuals were assigned to the Eastern breeding population.

**FIGURE 4:**
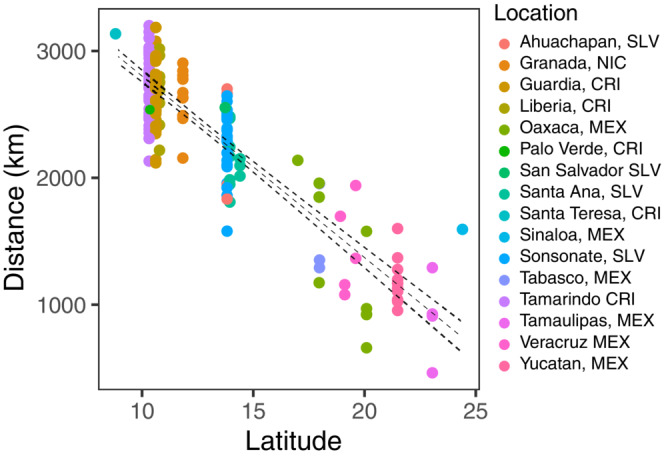
Correlation between predicted migration distance and predicted breeding latitude. Colors represent wintering locations.

Lastly, to better understand the observed migratory patterns in space, we estimated the straight‐line distance from the predicted breeding location to the wintering location of each individual. If Painted Buntings exhibit a leapfrog migration pattern, we would expect birds further north traveling to winter further south, thus traveling greater migration distances. Here we found that, indeed, individuals sampled at lower winter latitudes traveled greater distances (i.e., the predicted breeding area was further north; *p* > .001; Figure 4).

## 4 DISCUSSION

The ability to comprehensively characterize movement patterns in birds across time and space has been limited by technological approaches that allow for data collection from 100 of individuals. Here, we use a genoscape approach to identify genetically distinct groups within the species and then use the same genetic information to characterize migratory movements. Overall, the breeding genoscape supports the existence of four main groups within the species: Eastern, Southwestern, Central, and Louisiana (Figure 2). When genetic markers are further used to track the movement pattern of individual birds, we find strong support for the existence of a “Leapfrog” migration pattern.

### 4.1 Migratory connectivity in the Painted Bunting

Our results show that the Painted Bunting can be separated into four distinct genetic breeding populations—a Central, East Coast, Louisiana, and Southwest genetic group—rather than the three previously described by Battey et al. (2018) and Contina et al. (2019). The additional genetic structure detected here likely resulted from increased sampling density and an increase in the number of genetic markers used (over 10‐fold more than in Battey et al., 2018). Similar to Contina et al. (2019), we found two genetically distinct main groups that match the previously described subspecies—the eastern breeding populations, *P. c. ciris*, and the interior breeding populations, *P. c. pallidior* (Lowther et al., 2020). However, unlike previous studies, we found additional population structure within the Interior subspecies (e.g. Southwest, Louisiana, and Central). Further analysis of wintering samples across Mexico and parts of Central America identified a high degree of mixing between genetic groups within the Interior subspecies but no East Coast individuals. When our results are compared with recent connectivity work focused on the Eastern subspecies using light‐level geolocators (Sharp, 2021, Rushing et al., 2021), it becomes clear that the absence of East Coast individuals in our wintering samples is because they primarily winter in Cuba. Alternatively, our analysis of Spring and Fall migrants revealed that Central and Louisiana breeders overlap during spring along the Gulf Coast but only Central breeders migrate to western Mexico first before migrating south in the Fall. The presence of Central breeders in western Mexico that molt‐migration—a behavior where individuals first migrate to southern monsoon areas (Nayarit, Jalisco) to complete their molt before migrating further south to wintering areas (Contina et al., 2013)—may be unique to the Central group. Overall, our results suggest that migratory connectivity is strong between the Eastern and Interior subspecies groups but weaker within genetically distinct groups within the Interior subspecies.

The division between Eastern and interior subspecies has been previously described based on morphological (Storer, 1951) and life‐history differences (Pyle, 1997), as well as the species phylogeography (Herr et al., 2011), which concluded that the two‐known subspecies evolved separately from one ancestral taxon, likely from the range occupied by the current Southwestern genetic group. Our results support those of Battey et al. (2018) suggesting that the disjoint distribution, distinct migratory routes and overwintering locations (Sharp, 2021), and divergent molting strategies of Interior and Eastern breeding populations may be partially maintained by the existence of a migratory divide—defined as a region of contact between population with divergent migratory strategies. It is possible that the high cost of flying around or over the Gulf of Mexico results in fitness consequences for birds with intermediate migratory behavior, resulting in additional barriers to gene flow between the two forms. While further study is needed, it is possible that these Eastern and Western forms represent cryptic species (Johnson & Marten, 1988; Linck et al., 2019). Regardless this and other work strongly suggest that the two subspecies represent distinct Conservation Units and should be managed separately.

In addition to further supporting the division between Interior and Eastern subspecies, we also found the Southwest genetic group to be genetically distinct from the remaining populations. To our knowledge, this is the first study to report such a finding. This is particularly relevant given that previous studies (Contina et al., 2013; Herr et al., 2011) proposed that the current populations evolved in that area from a Mesoamerican ancestor and could explain why the inland western populations use a two‐step migration to winter further south, from Southeast Mexico through northern Panama. We did not pick up any individuals of the Southwest group on their wintering grounds, so it is unclear the extent to which Southwest breeders winter in distinct or overlapping locations with the other Inland breeders, but the observed genetic differences within the Southwest warrant further investigation from a conservation and management perspective.

### 4.2 Leapfrog migration in the painted bunting

Perhaps the most striking result from our individual‐level analysis of breeding origin was the highly significant relationship between migratory distance and wintering latitude in the west, indicative of leapfrog migration (Figure 4). A similar leapfrog pattern was also documented in the aforementioned geolocator study of the Eastern Painted Buntings. In this study, researchers demonstrated that a northern breeding population in North Carolina wintered further south in Cuba compared with southern breeding populations in Florida that were found equally in all nonbreeding ranges (e.g., south Florida, the Bahamas, and Cuba; Rushing et al., 2021; Sharp, 2021). This leapfrog migration has also been documented in other migratory species, such as Gambel's White‐crowned Sparrow (Lisovski et al., 2019) and more frequently in shorebirds (Boland, 1990; Kalejta & Hockey, 1994).

Suspected drivers of leapfrog migration include resource tracking, competition, and habitat quality (Newton, 2008). While reasons for the observed leapfrog patterns require further investigation, one idea is that they evolved as a means of reducing competition (Newton, 2008). Under the competition hypothesis, more‐northern breeding individuals may skip overwintering areas already occupied by resident populations or earlier arriving more‐southern breeders. In the Painted Bunting, southern breeders from the Central and Louisiana groups may leapfrog over sedentary species of the genus *Passeria* (*P. leclancheri* in central Mexico and *P. rositae* in southwestern Mexico) in southwestern Mexico. Alternatively, leapfrog migration may be explained by variations in habitat quality at breeding and wintering location. Specifically, high‐quality breeding habitats further north may support longer‐distance migration, allowing birds to overwinter further south where they may also have greater access to resources (Bell, 1997; McKinnon et al., 2015). The ability of individuals from the Central group to withstand longer migrations may be further supported by the molt‐migration strategy that allows them to take advantage of resource pulses in western Mexico before migrating south. Finally, the fasting endurance hypothesis posits that individuals that experience harsher conditions at the breeding site may be driven to compensate for those harsh conditions by migrating farther to reach highly favorable wintering conditions and in turn gain positive carryover to the breeding grounds (Chapman et al., 2011; Gow & Wiebe, 2014). Future work involving measuring fat loads and the quality of migrating individuals along their migratory journey may help distinguish between the different hypotheses to explain leapfrog migration. Overall, our results suggest that the Painted Bunting represents one of the clearest and most comprehensively document examples of leapfrog migration in nature.

### 4.3 Conclusions

We demonstrate that genetic tools combined with intensive sampling provide a powerful method for identifying range‐wide patterns of genetic and migratory connectivity across the range. Evidence of four distinct genetic groups within the Painted Bunting, with particularly strong migratory connectivity between East Coast and Interior populations, adds to our understanding of how to manage these populations in a rapidly changing world. Moreover, the existence of leapfrog migration within the Interior populations adds to the growing body of literature suggesting that spatial and temporal variation in resource abundance likely helps explain broadscale patterns of migratory connectivity. Future studies focused on testing hypotheses underlying the observed migratory patterns would further contribute to our understanding of the ecological process at work.

## AUTHOR CONTRIBUTIONS

**Rafael Rueda‐Hernandez:** Data curation (equal); formal analysis (equal); methodology (equal); writing – original draft (equal); writing – review and editing (equal). **Christen M. Bossu:** Data curation (equal); formal analysis (equal); methodology (equal); supervision (equal); validation (equal); visualization (equal); writing – review and editing (equal). **Thomas B. Smith:** Writing – review and editing (equal). **Andrea Contina:** Conceptualization (equal); visualization (equal); writing – review and editing (equal). **Ricardo Canales del Castillo:** Data curation (equal); investigation (equal); writing – review and editing (equal). **Kristen Ruegg:** Conceptualization (equal); data curation (equal); funding acquisition (equal); investigation (equal); methodology (equal); project administration (equal); resources (equal); supervision (equal); validation (equal); visualization (equal); writing – review and editing (equal). **Blanca Estela Hernandez‐Baños:** Conceptualization (equal); investigation (equal); supervision (equal); visualization (equal); writing – original draft (equal); writing – review and editing (equal).

## Supporting information


Appendix S1

## Data Availability

The genetic data and input files for population assignment to distinct genetic clusters using rubias and input files to estimate the geographic origin of individuals using OriGen can be found on dryad https://doi.org/10.5068/D1P96N.
