# Supplementary material for: Winter connectivity and leapfrog migration in a migratory passerine
Source: Ecol Evol. 2023 Feb 1;13(2):e9769. doi: 10.1002/ece3.9769 (PMC9891943; doi:10.1002/ece3.9769)
Supplement: Supplementary file 1 — Appendix S1 [file ECE3-13-e9769-s001.docx]

Supplemental Material.

Supplementary Tables.

Table S1. Number of Painted Buntings successfully screened using RADseq or SNP genotyping at each location across the species breeding, wintering and migratory range. Uppercase letters correspond to the map code of breeding populations in Fig. 2. Stage refers to when the individual was sample: breeding season (light grey), winter (dark grey), or sampled as a spring or fall migrant (intermediate grey).

| **Map code** | **Location** | **State** | **Country** | **Stage** | **Lat** | **Long** | **SNP type** | **RADseq** | **Sample type** |
| --- | --- | --- | --- | --- | --- | --- | --- | --- | --- |
| A | Big Bend | TX | USA | Breeding | 29.26 | -103.28 | 3 | 17 | Blood |
| B | Coahuila | Coahuila | MEX | Breeding | 27.46 | -101.33 | 17 | 0 | Feather |
| C | Artesia Wells | TX | USA | Breeding | 28.31 | -99.43 | 23 | 0 | Feather |
| D | Roswell | NM | USA | Breeding | 33.48 | -104.42 | 10 | 0 | Feather |
| E | Brownwood | TX | USA | Breeding | 31.65 | -98.92 | 26 | 0 | Feather |
| F | Nolanville | TX | USA | Breeding | 31.18 | -97.56 | 33 | 0 | Feather |
| G | Fairview | TX | USA | Breeding | 33.15 | -96.60 | 25 | 0 | Feather |
| H | Paducah | TX | USA | Breeding | 34.16 | -100.34 | 17 | 6 | Blood |
| I | Wichita | OK | USA | Breeding | 34.73 | -98.71 | 15 | 9 | Blood |
| K | Lawton | OK | USA | Breeding | 34.67 | -98.51 | 4 | 0 | Feather |
| L | Norman | OK | USA | Breeding | 35.23 | -97.35 | 4 | 16 | Blood |
| M | Stillwater | OK | USA | Breeding | 36.12 | -97.07 | 3 | 0 | Feather |
| N | Biological Station | OK | USA | Breeding | 33.88 | -96.80 | 5 | 10 | Blood |
| O | Deep Fork | OK | USA | Breeding | 35.53 | -95.92 | 4 | 0 | Blood |
| P | Okay | OK | USA | Breeding | 35.89 | -95.31 | 2 | 0 | Feather |
| Q | Sequoyah | OK | USA | Breeding | 35.43 | -94.99 | 3 | 13 | Blood |
| R | Holla Bend | AR | USA | Breeding | 35.13 | -93.07 | 4 | 13 | Blood |
| S | Red River | LA | USA | Breeding | 32.11 | -93.46 | 4 | 7 | Blood |
| T | Salt Lake | LA | USA | Breeding | 31.82 | -93.24 | 5 | 8 | Blood |
| U | St. Catherines Creek | LA | USA | Breeding | 31.32 | -91.44 | 0 | 10 | Blood |
| V | Point Coupee | LA | USA | Breeding | 30.50 | -91.74 | 2 | 0 | Feather |
| W | Bayou Cocodrie | LA | USA | Breeding | 29.25 | -90.66 | 2 | 7 | Blood |
| X | Bald Head Island | NC | USA | Breeding | 33.86 | -77.99 | 2 | 16 | Blood |
| Y | Isle of Hope | GA | USA | Breeding | 31.88 | -80.98 | 40 | 0 | Feather |
| Z | Ormond Beach | FL | USA | Breeding | 29.35 | -81.09 | 1 | 0 | Feather |
|  |  |  |  |  |  | **Total Breeding** | | **386** |  |
| - | Edomex | Edomex | MEX | Fall Migrant | 18.48 | -98.98 | 1 | 0 | Feather |
| - | Guerrero | Guerrero | MEX | Fall Migrant | 17.02 | -99.77 | 10 | 0 | Tissue |
| - | Sinaloa | Sinaloa | MEX | Fall Migrant | 24.86 | -107.35 | 7 | 0 | Feather |
| - | Sonora | Sonora | MEX | Fall Migrant | 26.93 | -108.69 | 11 | 0 | DNA |
| - | Yucatan | Yucatan | MEX | Fall Migrant | 21.49 | -87.53 | 12 | 0 | Feather |
| - | Bastrop | TX | USA | Fall Migrant | 30.28 | -97.31 | 2 | 0 | Feather |
| - | Cameron | TX | USA | Fall Migrant | 26.24 | -97.58 | 1 | 0 | Feather |
| - | Fairview | TX | USA | Fall Migrant | 33.15 | -96.60 | 7 | 0 | Feather |
| - | Indian_Creek | TX | USA | Fall Migrant | 31.60 | -98.92 | 2 | 0 | Feather |
| - | Ormond Beach | FL | USA | Fall Migrant | 29.22 | -81.06 | 4 | 0 | Feather |
| - | Roswell | NM | USA | Fall Migrant | 33.48 | -104.42 | 8 | 0 | Feather |
| - | Sinton | TX | USA | Fall Migrant | 28.10 | -97.38 | 1 | 0 | Feather |
| - | Stillwater | OK | USA | Fall Migrant | 36.12 | -97.07 | 1 | 0 | Feather |
| - | Guardia | Guanacaste | CRI | Spring Migrant | 10.62 | -85.62 | 32 | 0 | Feather |
| - | Puntarenas | Costa Rica | CRI | Spring Migrant | 8.80 | -82.96 | 2 | 0 | Feather |
| - | Santa Teresa | Costa Rica | CRI | Spring Migrant | 8.81 | -82.92 | 1 | 0 | Feather |
| - | Tamarindo | Guanacaste | CRI | Spring Migrant | 10.32 | -85.83 | 22 | 0 | Feather |
| - | Santa Ana | El Salvador | SLV | Spring Migrant | 13.94 | -89.62 | 9 | 0 | Feather |
| - | Sonsonate | El Salvador | SLV | Spring Migrant | 13.82 | -89.65 | 6 | 0 | Feather |
| - | Morales | Izabal | GTM | Spring Migrant | 15.64 | -88.87 | 1 | 0 | Feather |
| - | Coahuila | Coahuila | MEX | Spring Migrant | 27.89 | -101.14 | 1 | 0 | Feather |
| - | Veracruz | Veracruz | MEX | Spring Migrant | 19.27 | -96.19 | 16 | 0 | Feather |
| - | Bastrop | TX | USA | Spring Migrant | 30.27 | -97.29 | 6 | 0 | Feather |
| - | Flat | TX | USA | Spring Migrant | 31.36 | -97.66 | 2 | 0 | Feather |
| - | Fort Hood | TX | USA | Spring Migrant | 31.25 | -97.61 | 1 | 0 | Feather |
| - | Killeen | TX | USA | Spring Migrant | 31.18 | -97.62 | 1 | 0 | Feather |
| - | Mad Island | TX | USA | Spring Migrant | 28.63 | -96.12 | 1 | 0 | Feather |
| - | Ormond Beach | FL | USA | Spring Migrant | 29.26 | -81.07 | 3 | 0 | Feather |
| E | Brownwood | TX | USA | Spring Migrant | 31.64 | -98.94 | 2 | 0 | Feather |
| F | Nolanville | TX | USA | Spring Migrant | 31.18 | -97.56 | 3 | 0 | Feather |
| G | Fairview | TX | USA | Spring Migrant | 33.15 | -96.60 | 54 | 0 | Feather |
|  |  |  |  |  |  | **Total Migrant** | | **230** |  |
| - | Guardia | Guanacaste | CRI | Winter | 10.62 | -85.62 | 45 | 0 | Feather |
| - | Liberia | Guanacaste | CRI | Winter | 10.78 | -85.67 | 10 | 0 | Feather |
| - | Palo Verde | Guanacaste | CRI | Winter | 10.35 | -85.35 | 1 | 0 | Feather |
| - | Tamarindo | Guanacaste | CRI | Winter | 10.32 | -85.83 | 41 | 0 | Blood, Feather |
| - | Ahuachapan | El_Salvador | SLV | Winter | 13.82 | -89.94 | 7 | 0 | Feather |
| - | San Salvador | El_Salvador | SLV | Winter | 13.74 | -89.29 | 1 | 0 | Feather |
| - | Santa Ana | El_Salvador | SLV | Winter | 14.40 | -89.36 | 3 | 0 | Feather |
| - | Sonsonate | El Salvador | SLV | Winter | 13.82 | -89.65 | 29 | 0 | Feather |
| - | Oaxaca | Oaxaca | MEX | Winter | 18.89 | -98.16 | 14 | 0 | Blood, Feather |
| - | Sinaloa | Sinaloa | MEX | Winter | 24.40 | -106.61 | 1 | 0 | Feather |
| - | Tabasco | Tabasco | MEX | Winter | 17.97 | -92.80 | 4 | 0 | Feather |
| - | Tamaulipas | Tamaulipas | MEX | Winter | 23.05 | -99.15 | 4 | 0 | Feather |
| - | Veracruz | Veracruz | MEX | Winter | 19.44 | -96.29 | 3 | 0 | Feather |
| - | Yucatan | Yucatan | MEX | Winter | 21.49 | -87.53 | 5 | 0 | Feather |
| - | Granada | Nicaragua | NIC | Winter | 11.83 | -86.01 | 9 | 0 | Blood, Feather |
| - | Rivas | Nicaragua | NIC | Winter | 11.20 | -85.65 | 1 | 0 | Feather |
|  |  |  |  |  |  | **Total Winter** | | **178** |  |

Table S2. Assignment of Painted Buntings of known origin back to distinct genetic clusters using the program *rubias*. The colors indicate the predicted genetic group of origin (Fig. 2). Accuracy is based on the percentage of correct assignments.

| **Distinct breeding unit** | **Southwest** | **Central** | **Louisiana** | **East coast** | **Total Number** | **Accuracy** |
| --- | --- | --- | --- | --- | --- | --- |
| **Southwest** | **0** | 3 | 0 | 0 | **3** | **0** |
| **Central** | 2 | **194** | 4 | 0 | **200** | **0.97** |
| **Louisiana** | 0 | 7 | **7** | 0 | **14** | **0.5** |
| **East coast** | 0 | 0 | 0 | **43** | **43** | **1** |

Supplementary Figures

Figure S1. Population Structure in Painted buntings assuming different numbers of populations. Ancestry plots generated in *structure* corresponding to K values of 2 – 5, illustrating support for 4 genetically distinct lineages within Painted buntings. The sampling locations along the bottom bar correspond to the breeding locations illustrated in Figure 2 and Table S1. The Evanno method plot demonstrates a plateau at K=4 (B), suggesting K=4 is the optimal number of genetic clusters.
